# Reviewing the burden of comorbidity in patients receiving specialist in-patient treatment for drug and alcohol problems

**DOI:** 10.1192/bjb.2020.4

**Published:** 2020-08

**Authors:** Alice Bradley, Amy Martin

**Affiliations:** 1 Peninsula College of Medicine and Dentistry, UK; 2 University of Aberdeen, UK

**Keywords:** Alcohol disorders, clinical governance, comorbidity, drugs of dependence disorders, in-patient treatment

## Abstract

**Aims and method:**

To compare and contrast the burden of comorbidity in a population receiving in-patient treatment for substance misuse with that of a cohort admitted to the same unit 4 years previously. The Charlson Comorbidity Index (CCI) was used to quantify patients' comorbidity and predict 10-year survival.

**Results:**

There was a marked reduction in predicted 10-year survival: in 2014, 22% of patients had a predicted 98% chance of 10-year survival, whereas only 2% in the 2018 cohort had a predicted 98% chance. Additionally, in 2014 only 9% of patients had a <20% 10-year predicted survival chance, whereas 28% in 2018 had a predicted 10-year survival chance of <20%. In this time, funding for services was cut by 23% and the 12-bed unit was reduced to 8 beds. This resulted in an increase in the average waiting time from 30 to 65 days. In 2018, more patients were admitted for alcohol detoxification, rising from 79% to 93% of admissions. Chronic respiratory disease remains the most prominent comorbidity; however, there is also an increase in the percentage of patients with liver disease.

**Clinical implications:**

In-patient substance misuse units are known to serve individuals with complex illnesses. With service funding cuts, subsequent bed reductions and increased waiting times, this complexity is increasing, with a considerably higher burden of comorbidity. The consequential increased mortality risk highlights the ongoing need for adequate community and in-patient services with integrated care of mental and physical health alongside social work.

It is widely accepted that there are social, psychological and physical harms associated with chronic substance misuse.^1^ It has also been identified that drug and alcohol misuse are independent risk factors for physical illness in people with pre-existing mental illness.^2^ Furthermore, the World Health Organization (WHO) found alcohol consumption to be the third highest risk factor, in high-income countries, for disease burden, superseded only by smoking and hypertension.^3^ Specialist in-patient units offer drug and alcohol detoxification and stabilisation to individuals with complex problems, whose substance misuse needs cannot be met in the community. Despite this, there have been significant funding cuts for alcohol and drug prevention, treatment and support services in Scotland in recent years.^4^ This study seeks to describe and evaluate the burden of comorbidity carried by a current population of in-patients in a substance misuse unit in Edinburgh. It was anecdotally noted among the staff of this unit that in-patients were requiring more medical intervention. We therefore sought to compare the disease burden of a patient cohort in 2018 with that of a 2014 cohort in order to identify any developments in the prevalence or character of comorbidity carried by this population. These data provide invaluable insight into a high-risk population and aim to recognise any areas of developing trends within this group, to identify possible areas for intervention in the future.

## Method

We conducted a retrospective study of in-patients over a 6-month period in 2018. We sought to explore the population's characteristics and compare these with the data collected by Mogford & Lawrence for their primary study conducted in the same unit in 2014.^5^ To quantify comorbidity, cumulative disease burden scales are used. These are calculated by attributing scores to specific diseases, health behaviours and medications, which are weighted according to their impact on morbidity and mortality. These scores are combined to assign an overall index of comorbidity, which can be used to compare, contrast and track change in individual patients or patient groups. The Charlson Comorbidity Index (CCI) has previously been used across a wide range of clinical settings and a variety of patient populations, including the 2014 cohort. To allow a direct comparison of patient cohorts, the same CCI was implemented to quantify comorbidity in the repeat patient cohort in 2018. There is a strong base for using the CCI, which has been deemed valid and reliable for estimating the burden of comorbidity in clinical research.^6^ The index records 16 diagnoses, including chronic respiratory disease and congestive heart failure. These details are all obtained as part of the routine documentation completed on admission to the unit. These 16 diagnoses have validated predictive value when calculating projected 10-year survival. The impact of age on predicted survival is accounted for by the addition of an age-based score to the raw CCI. The version of the CCI used is an extended one that includes four additional items that predict healthcare cost, but are excluded from the calculation of predicted 10-year survival.^7^ This version held the additional benefit of including a greater breadth of conditions/factors, specifically hypertension, skin ulcers or recurrent cellulitis, treatment with warfarin and the presence of depression. The individual patient scores could then be used to calculate their predicted 10-year survival.

We assert that all procedures contributing to this work comply with the ethical standards of the relevant national and institutional committees on human experimentation and with the Helsinki Declaration of 1975, as revised in 2008. Ethics approval was not required.

### Participants

There were a total of 138 participants, who were admitted over a 6-month period to the Ritson Clinic at the Royal Edinburgh Hospital in Scotland. All the patients who were admitted to the unit were included in the data-set. The Ritson Clinic is a specialist unit that provides in-patient detoxification, stabilisation and replacement therapy for people with alcohol, opiate and other drug dependencies such as benzodiazepines, stimulants and novel psychoactive substances. All of the patients have complex psychiatric and social needs that are best met in an in-patient setting. Referrals are received from a range of community teams, including general practice and substance misuse services as well as some acute services such as the regional infectious diseases unit. These are all within the local National Health Service (NHS) health board of Edinburgh and the Lothians, with a population of over 800 000. The participants included in the study were ‘recruited’ retrospectively from the 6-month period immediately prior to the start of data collection. The period covered was from 19 April to 22 October 2018. The original study recruited patients from 26 May to 27 November 2014 in a prospective manner.^5^

### Data collection

All collected data had been gathered during the in-patient admission clerking that was part of routine practice at the unit. As with the data collection in 2014, a brief paper recording tool was used for standardised collection of data. The authors, A.B. and A.M., completed the brief paper recording tool, retrospectively, that was created for completion during admission in the 2014 study. This information included a full psychiatric history, substance misuse history, medical history, physical examination and appropriate investigations, including a routine set of blood tests. To provide collateral information, a full range of data sources were used to complete the summary. These sources included a verbal report from the patient, electronic patient records, out-patient letters, previous discharge letters and general practitioner clinical summaries. The CCI provides detailed and specific descriptions of the inclusion criteria for each diagnosis, allowing for objectivity and repeatability in assessment so that each diagnosis was given the appropriate score. This data collection was conducted in the same manner as the data collected from 2014 cohort had been, as provided by Mogford & Lawrence, to allow for direct comparison.^5^

## Results

In total, 138 patients were admitted during the repeat 6-month period of data collection. This compares with 175 patients admitted during the original period of data collection in 2014. The profile, number of patients admitted for alcohol detoxification, substance misuse diagnosis, the presence of a diagnosis and prevalence of cigarette smoking are shown in Table 1. The average age of patients for treatment has increased from 44 years to 49 years. There has been an increase, from 80% to 93%, in the number of patients who were admitted for alcohol detoxification. There has been a slight fall, from 80% to 76%, in the number of patients who were smokers at the time of admission. At the time of data collection for the 2018 study, 14% of the 2014 cohort was deceased and 2% of the current cohort was already deceased.

**Table 1:** Study group characteristics

	2018 cohort	2014 cohort
Age, years: mean (range)	49 (23–70)	44 (19–73)
Male, *n* (%)	60 (43)	111 (63)
Female, *n* (%)	78 (56)	64 (37)
Patients admitted for alcohol detoxification, *n* (%)	128 (93)	139 (80)
Comorbid depression diagnosis, *n* (%)	100 (72)	82 (47)
Cigarette smoker, *n* (%)	105 (76)	138 (80)
Patients deceased at 2018 data collection point, *n* (%)	3 (2)	25 (14)

**Fig. 1:**
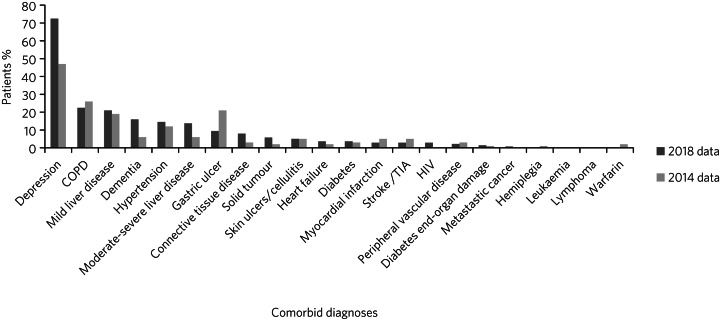
Comparison of comorbid diagnoses/factors recorded for the 2014 and 2018 study cohorts. COPD, chronic obstructive pulmonary disease; TIA, transient ischaemic attack.

The proportions of patients with each of the CCI items are summarised in Fig. 1. Within both cohorts, the condition with the highest prevalence was depression. Of note, however, this has shown a marked increase, from 47% in 2014 to 72% in 2018. Depression is the only psychiatric condition included in the CCI. There was a small reduction in the percentage of patients with chronic obstructive pulmonary disease (from 26% in 2014 to 22% in 2018), which we would expect with the reduction seen in the percentage of smokers over this period. In the 2018 cohort there is an increase in prevalence of both mild liver disease (from 19% to 21%) and moderate to severe liver disease (from 6% to 14%). This is in the context of an increased number of admissions for the purpose of alcohol detoxification. There is also a marked increase in the percentage of patients who met the criteria for diagnosis of dementia (from 6% in 2014 to 16% in 2018).

Table 2 presents the comparison of the distribution of CCI scores and associated 10-year mortality. The percentage of patients with a predicted 10-year survival chance <20% has increased from 9% to 28%. In 2014, 63% of patients had a predicted 10-year survival chance >90%, whereas in 2018 this was only 16%.

**Table 2:** Comparison of Charlson Comorbidity Index (CCI) scores and predicted 10-year survival for 2018 and 2014 cohorts

CCI score	Predicted 10-year survival chance	2018, *n* (%)	2014, *n* (%)
>5	<20%	38 (28)	16 (9)
5	20–49%	17 (12)	13(7)
4	50–74%	24 (17)	12 (7)
3	75–90%	37 (27)	24 (14)
<3	>90%	22 (16)	110 (63)

**Fig. 2:**
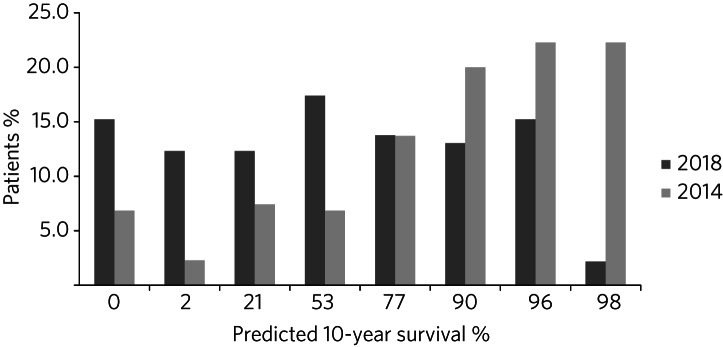
Comparison of predicted 10-year survival for the 2014 and 2018 study cohorts.

**Fig. 3:**
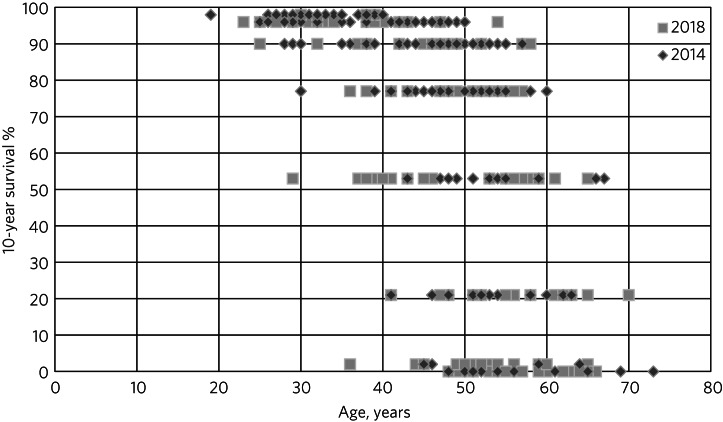
Comparison of age and predicted 10-year survival for the 2014 and 2018 study cohorts.

As shown in Fig. 2, in 2014 only 7% of patients were predicted to have no chance of survival at 10 years, compared with 15% in 2018. There were just 2% of patients with a 10-year predicted survival chance >98% in 2018, whereas this was 22% in 2014. The interaction between age and predicted survival is demonstrated in Fig. 3. The number of younger patients carrying a high burden of comorbidity has increased.

## Discussion

### The Ritson Clinic and government policy on drug and alcohol services

The Royal Edinburgh Hospital site is currently being redeveloped, with the Ritson Clinic being one of the last areas of the hospital to undergo service redevelopment. This has led to considerable discussion about whether the unit would be best placed at a medical hospital or on a psychiatric site and about level of intervention the in-patients require. In addition, on a wider scale, there is ongoing debate regarding the allocation of resources to fund specialist in-patient treatment of drug and alcohol problems within NHS Lothian. The original study^5^ helped make the case for the ongoing availability of a medically supported in-patient unit for the treatment of alcohol and drug use disorders within NHS Lothian. In the intervening period, service pressures have increased and available bed numbers have decreased. Between 2016 and 2017 direct Scottish Government funding for alcohol and drug partnerships (ADPs, local partnerships between health boards, local authorities, police and voluntary agencies to tackle alcohol- and drug-related problems) fell by 22%.^4^ In Lothian, the ADP funding allocations for alcohol and drug prevention, treatment and support services was cut from £11 469 680 in 2015–2016 to £8 887 133 in 2016–2017.^8^ As a consequence of this reduction in national funding, by January 2018 the number of beds in the Ritson Clinic was reduced from 12 to 8. These changes have occurred in the context of changes in public health policy, which increasingly focuses on the preventability of drug- and alcohol-related deaths. The average waiting time for an admission to the Ritson Clinic has increased from 30 days for the 2014 cohort to 65 days in 2018. The average length of admission was 8.9 days for the 2018 cohort. The unit receives referrals from community addiction services, general practices (including practices for those without fixed accommodation), in-patient and out-patient hepatology teams, alcohol liaison nurses and the regional infectious diseases unit. These are screened using admission criteria originally based on Scottish Intercollegiate Guidelines Network (SIGN) guidelines.^9^ This ensures that admission for in-patient treatment is limited to those who could not have their needs met in the community; by its very nature, this predisposes them to carry a higher burden of comorbidity.^8^ It is worth noting that there are other factors that may contribute to increased morbidity in this patient group that were not accounted for in the study, such as poor nutrition and blood-borne viruses such as hepatitis B or hepatitis C. This study did, however, look at the prevalence of smoking in the patient group. Evidence suggests that the prevalence of smoking in the Scottish population is falling. In 2017, it was estimated that 18% of the Scottish adult population smoked.^10^ Despite showing a small improvement between 2014 and 2018, the in-patients included in our study still have a considerably increased prevalence of smoking; 76% of the 2018 cohort were current smokers, which will contribute to their overall increased morbidity and mortality.

### Reductions in 10-year predicted survival

Data from the Office for National Statistics for 2011–2013 estimated the predicted 10-year survival chance for a 45-year-old Scottish male to be 96.6%.^11^ Comparatively, in the original cohort, among patients aged between 45 and 55, the mean predicted 10-year survival chance was 68.6%. In the 2018 cohort, this had reduced to 55%. There are multiple likely contributing factors to the increase in predicted mortality over the 4 years. The increased waiting times and reduced bed numbers have certainly played a role. When screening referrals to the unit, the patients with the greatest need are given preference, with those with highest immediate risk to physical well-being taking precedence. This is also likely to explain why there is an increase in the number of patients admitted for treatment of alcohol dependence over the 4 years, as the risk to physical health from alcohol withdrawal is usually greater than that of drug stabilisation or detoxification, therefore these patients are admitted preferentially. As per the SIGN guidelines any patient who is confused, has a history of seizures or hallucinations, has an acute physical or psychiatric illness, including multiple substance misuse, has previously failed home-assisted detoxification or has a home environment unsupportive of abstinence is deemed to require in-patient detoxification.^9^ These broad inclusion criteria cover much of the patient population that would require alcohol detoxification and also go some way to explain why the large majority of admissions, in both 2014 and 2018, were for treatment of alcohol dependency, despite the Ritson being a unit that treats both drug and alcohol problems. With increased waiting times and pressure on beds, the triage of referrals requires ever more challenging clinical decision-making.

### Increasing burden of comorbidity

The Ritson Unit currently forms part of the Royal Edinburgh Hospital site, which provides only psychiatric care and is not a medical hospital. Anecdotally, among the multidisciplinary team, it was felt that the patients being admitted to the unit had increasingly complex medical problems, requiring more medical intervention beyond the capabilities of the unit. Patients admitted to the unit who become acutely medically unwell often require transfer across the city to the medical hospital, as their physical health needs cannot be safely managed within the psychiatric hospital setting. Our data have supported this impression that patients are indeed carrying a higher burden of comorbidity. In quantifying this burden, the study demonstrates the ongoing and growing demand for in-patient facilities to treat this unique population of patients, whose needs intersect mental and physical healthcare services. To improve outcomes for these patients, a more collaborative and proactive approach in the development of these services is required. This is in accordance with the recommendation of increased funding and an emphasis on better integrated care made by NHS England in its NHS Long Term Plan.^12^

### Limitations

The study was conducted using the CCI, which is a useful tool for comparison of predicted mortality. We have compared two groups, admitted over the same length of time. However, because the capacity of the unit was reduced between the two studies, we compared a smaller, of an already small, sample (138 patients in 2018, compared with 175 in 2014). This meant that we used percentages for data analysis rather than gross numbers of patients. The CCI does not give an indication of the impact of morbidity on quality of life, nor does it demonstrate causation. A further limitation of the study is that the data were collected retrospectively. The initial study was carried out prospectively and by different clinicians, so there may have been a resultant difference when scoring the patients. However, the CCI is based on objective measures, so variability should be limited. The score is predicted over 10 years, and we have repeated the study after 4 years. We are therefore unable to compare the predicted mortality with the actual mortality. Furthermore, one cannot extrapolate the current number of deceased patients to draw any conclusions about the accuracy of the scoring in this patient group. In addition, although the score demonstrates a declining trend in health outcomes in this patient population, it does not provide any indication of quality of life or functionality of patients.

### Implications

The original data collected in 2014 showed a high burden of physical illness carried by those admitted for in-patient treatment for substance misuse. The data collected in 2018 confirm what was suspected from observations made by the healthcare professionals working within the service: that patients being admitted have an increasing burden of comorbidity, requiring more intervention from specialties, and ultimately are likely to have worse long-term health outcomes. At 4 years, 14% of patients originally audited were deceased. It is recommended that the study is repeated after 10 years, to assess the accuracy of the score at predicting mortality in this population with complex, specialist care needs. The intersection between physical healthcare requirements and psychiatric and substance misuse needs continues to present a unique challenge in caring for this group of patients. Although the current trend is to move towards a more community-based healthcare model, the increased comorbidity and growing waiting lists identify the ongoing need, within this population, for in-patient services. There is clearly a requirement for healthcare providers and facilities that can provide safe and effective treatment of the combined conditions and substance misuse needs of these patients. Quantifying the degree of comorbidity within this population remains valuable for the allocation of resources and development of services with an emphasis on integrated care.

## About the authors

**Alice Bradley**, BMBS (Peninsula College of Medicine and Dentistry) is a Junior Clinical Fellow in general surgery at NHS Fife, and she was a foundation year 2 (FY2) doctor in NHS Lothian at time of writing. **Amy Martin**, MBChB (University of Aberdeen) is a general adult higher psychiatry trainee in NHS Lothian (ST4) and was a core psychiatry trainee (CT3) at time of writing.
